# Nondestructive Intervention to Multi-Agent Systems through an Intelligent Agent

**DOI:** 10.1371/journal.pone.0061542

**Published:** 2013-05-02

**Authors:** Jing Han, Lin Wang

**Affiliations:** 1 Key Laboratory of Systems and Control, Academy of Mathematics and Systems Science, Chinese Academy of Sciences, Beijing, China; 2 Department of Automation, Shanghai Jiao Tong University, Shanghai, China; Universidad de Zarazoga, Spain

## Abstract

For a given multi-agent system where the local interaction rule of the existing agents can not be re-designed, one way to intervene the collective behavior of the system is to add one or a few special agents into the group which are still treated as normal agents by the existing ones. We study how to lead a Vicsek-like flocking model to reach synchronization by adding special agents. A popular method is to add some simple leaders (fixed-headings agents). However, we add one intelligent agent, called ‘shill’, which uses online feedback information of the group to decide the shill's moving direction at each step. A novel strategy for the shill to coordinate the group is proposed. It is strictly proved that a shill with this strategy and a limited speed can synchronize every agent in the group. The computer simulations show the effectiveness of this strategy in different scenarios, including different group sizes, shill speed, and with or without noise. Compared to the method of adding some fixed-heading leaders, our method can guarantee synchronization for any initial configuration in the deterministic scenario and improve the *synchronization level* significantly in low density groups, or model with noise. This suggests the advantage and power of feedback information in intervention of collective behavior.

## Introduction

Multi-agent methodology is a natural and popular way to model a system consisting of many locally interacting individuals (units). Collective behavior such as phase transition, flocking/schooling/herding [1], pattern formation, swarm intelligence [2], synchronization [3] [4] [5], crowd panic [6], locust collective motion [7] and group decision [8], are widespread and abundant at the macroscopic level of multi-agent systems.

In a distributed multi-agent system, there is no central controller. Usually agents interact locally. The set of rules (or mechanisms) describing the interaction between two agents are called ‘local rules’. If the local rule is elaborately designed, the system will show expected and useful function, such as swarm intelligence. However, for some systems, the self-organized collective behavior is not what we expect. Then, how do we intervene in the system and change the collective behavior? One way is to re-design the multi-agent system. For example, re-design the local rule of the agents. That is actually about the problem of how to design a distributed system. Examples include formation control [9] for robots, ant colony algorithm [10] and distributed algorithm for constraint satisfaction problems [11], etc. By doing this, the system will self-organize to the desired collective behavior. Another method is to put some special agents into the system to coordinate the collective behavior, such as leaders with stronger influence [12], virtue leaders [13] and mediators [14], etc. Both of these methods require additional abilities of the existing normal agents to recognize and interact with the special ones.

The other way is to use nondestructive intervention methods. In many real world systems, such as birds and crowds, the interaction rules among individuals are part of the natural mechanism. They can not be re-designed to achieve the desired collective behavior. Therefore, the coordination should not change the interaction rules of the existing agents (normal agents). Meanwhile, in these decentralized systems there is no central controller who sends orders to agents, nor can global parameter be adjusted to change the collective behavior either. In this case, how do we softly intervene in the system and guide the collective behavior?

For some multi-agent systems, adding a few agents into the system is allowed. One way to intervene in the system is to add one (or more) special agent(s), called a ‘***shill***’, which is treated as a normal agent by normal ones. The shill is not like the stronger influential leader [12], virtue leader [13] or mediator [14] which should be treated differently by normal agents. The influence of a shill and that of a normal agent are equal in strength, while stronger influential leaders or mediator have more influence on others than normal agents. Thus, by adding a shill, influence is ‘softly’ put on the system, which is called ‘soft-control’ [15] [16]. Examples of such include adding some shills to promote cooperation in the population playing repeated Prisoner Dilemma games [17], or using ad hoc team agent(can be regarded as a shill) as a teacher to lead the other agent in the multi-armed bandit problem to maximize team utility [18], etc.

Synchronization is one of the most basic yet important collective behavior, which has profound impacts on many systems [3] [4]. A related classical model for studying synchronization, the Vicsek's model [19] (including its variant MAS models) is widely studied by physicists, mathematicians and control scientists in the current decade [20]–[32]. In these models, each agent moves with a constant speed and its heading is updated based on the average headings of its neighbors (including itself). It is a simple but nontrivial model. It display a rich set of phenomena, such as flocking/schooling/herding behavior, strong coupled and dynamical interaction networks, and the phase transitions of synchronization.

In situations where the initial configuration of the system does not satisfy the synchronization condition, intervention is needed to help synchronize the system – shills are added into the system. There are four related approaches: (1)For the Vicsek's model without noise, an early attempt to intervene and guide synchronization is described in [15] [16], where a shill with a fixed desired heading is simply placed near the agent with the worst heading at every time step. Then the system can converge to the desired heading asymptotically. (2)Later in [30], a number of leaders (fixed-heading shills with desired moving direction) are randomly distributed among the group of normal agents inside the initial area. Leaders simply move with a fixed heading forever and they have impact on their neighboring normal agents. They provide a theoretical proof of the proportion of leaders needed to guide a group to synchronization almost surely when the group size is large enough. (3)For the linear-Vicsek's model without noise, Jadbabaie et al. [20] added a special agent (fixed-heading shill with desired moving direction) to guide the group. They point out that the key is to maintain the connectivity of union of neighboring graphs consisting of all agents within some contiguous and bounded time intervals. However, they did not provide the algorithm of how the special agent moves to guarantee such connectivity. (4)Based on a different flocking model, Couzin et al. [8] studied how a few informed individuals (who know the direction to a resource) influence the moving decision of animal groups. They reveal that the larger the population the smaller the proportion of informed individuals that is required to guide the group by simulations.

In the above approaches, shills of [15] [16], informed individual [8], leader [30] and special agent [20], are all ***fixed-heading shills***. These shills are too simple because they do not use online feedback information to adjust their moving direction. A simple shill has limited intelligence and power. Therefore, one simple fixed-heading shill is unlikely to guide the group to synchronization. With more shills, the system is more likely to become synchronized. For this reason papers [8] and [30] focus on the number of shills. Their method only works in the probabilistic scenario for high density groups for the Vicsek's model. This is because once a normal agent moves outside the neighborhood of fixed-heading shills, it will never be affected in the future. The method in [15] can promise synchronization for any initial configurations in the deterministic scenario, and only one shill is needed. However, although the heading of the shill is fixed, it is not moving in a fixed direction. Actually it is arbitrarily placed in different target positions during evolution. Although online feedback information of locations and headings of agents is exploited by the shill, it is only used for the selection of target positions, not for the decision-making of the schematic movements of the shill. It does not solve the basic problem of how a shill moves from one location to another without putting negative effects (i.e., drawing heading of agents to a non-desired direction) on the group. There is no proof for the shill speed limitation and its moving direction is not consistent with the heading of the shill. Therefore, it is not a complete algorithm for intelligent shills.

In this paper, a comprehensive algorithm for an efficient and intelligent shill is introduced: it has a new and subtle strategy called ‘consistent moving’, which uses online information of normal agents' locations to determine and update its heading at each step. The shill periodically affects every normal agent and will eventually synchronize the whole group towards the desired heading. Both the mathematical analysis and the simulation results prove that the system can be synchronized by adding one intelligent shill with a limited speed. Merits of this new approach are as follow:

1. synchronization is guaranteed for any initial configuration in the deterministic scenario by adding only one intelligent shill;
2. the strategy is much more clever and the heading of the shill is consistent with its actual moving route, so this is the first comprehensive approach for intelligent shill with theoretical analysis;
3. by using feedback information, the intelligent shill is possible to handle noise in the Vicsek's model, which cannot be achieved with fixed-heading shills.

We will demonstrate these advantages in the computer simulations by comparing with the method of adding some fixed-heading shills (leaders). It shows that one intelligent shill can perform better when measured by the synchronization level, especially in low density groups. Besides, the intelligent shill has significant advantages in the case of noise. It implies that feedback information is essential for intervention in the model with noise.

For nondestructive intervention of collective behavior of multi-agent systems, adding shills is a feasible method. Although shills have only the same strength of influence on neighbors as normal agents do, they can have a bigger impact on the collective behavior of the group, depending on the number of shills that are added into the system and how intelligent the shills are. Without using feedback information, shills are not intelligent and more than one shill is needed to guide the group [8] [30]. Our approach in this paper confirms shills can become more powerful with feedback information. One intelligent shill is able to guide the whole group, and it can also handle more complicated cases.

## Methods

### The Multi-agent model

Flocking of birds, schooling of fishes and herding of sheep are ubiquitous in nature. In 1987, Reynolds, a computer scientist, might be the first to propose a computer simulation which can show flocking phenomena [1]. It is a multi-agent system with three simple local rules for each interacting agent: Alignment, Separation and Cohesion. Later in 1995, from a different viewpoint physicists Vicsek et al. introduced a simplified model, which only keeps the Alignment rule - steer towards the average heading of neighboring agents. Two agents are neighboring to each other if the distance between them is not larger than a given constant 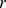. In spite of its simplicity, the self-order motion, a flocking-like cooperative behavior, emerged in this simplified system. Although in reality the physical and biological systems are more complicated, many of them are based on this model more or less [33]–[35].

The Vicsek-like model we adopt in this paper is described bellow: there are 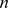 agents all moving simultaneously in the unlimited two-dimensional space. They are represented by heading set 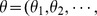 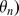 and location set 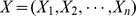, where 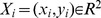 denotes the 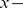
coordinate and the 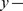
coordinate of the location of agent 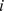. The velocity of agent 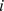 at time 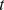 is 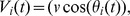 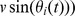, constructed to have a constant speed 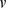 and a heading given by the angle 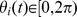. Neighbors of agent 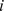 at time 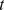 are those agents which are either in or on a circle of a constant radius 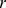 centered at agent 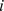
's current position. So the neighborhood is defined as
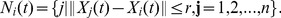
(1)


Agent 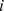 updates its heading and position according to equations as follow:
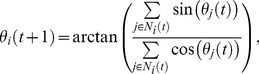
(2)

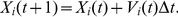
(3)where 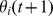 is the angle of the sum of the velocity vector of all neighboring agents, and the time unit 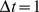 is the time interval between two updates.

### Adding an intelligent shill

**Figure 1:**
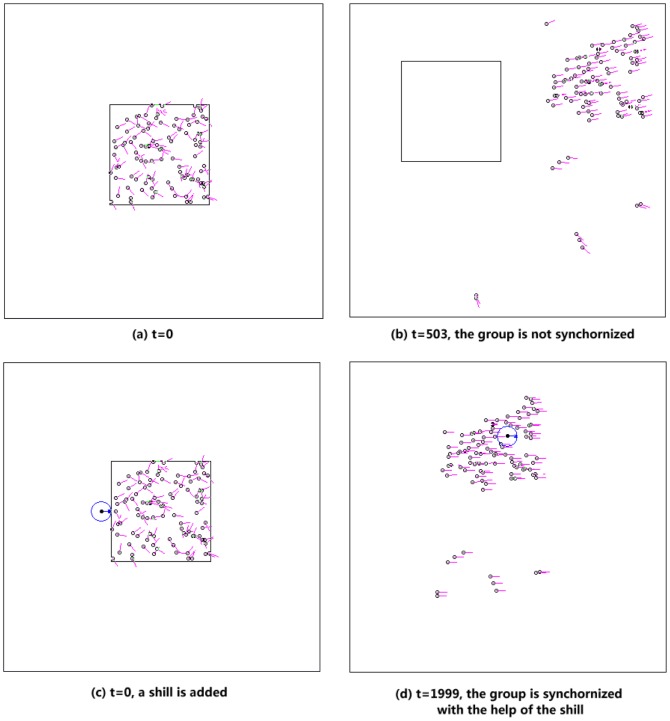
Snapshots of computer simulation. Velocities of agents are displayed for two cases: a self-organized group and a group with one shill. The number of agents is 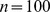 in each case. Normal agents are represented by the little circle with red line pointing to the moving direction. (a) and (b) are for the self-organized case. (a) 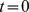, 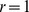, 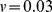. 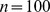 agents are randomly distributed inside a square with 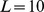. (b) After 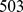 steps, the system reaches to a situation of several separated subgroups with different moving directions. So the group does not self-organize to a same heading -synchronization. (c) and (d) are for the case with a shill: (c) 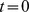, a shill with 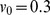 is added into the same group as shown in (a). The shill is denoted as a dark little dot with blue arrow pointing to its heading direction. The blue circle centered at it indicates its neighborhood area (the radius is 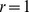, which is the same as the radius of normal agents). (d) With the help of the shill using ‘consistent moving’ strategy, after 1999 steps, headings of all agents converge to a desired heading (zero), i.e., 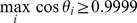.

The computer simulation of the above model shows that given different initial configurations (initial locations and headings of 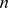 agents), the system will exhibit different phenomena: agents evolve to a same heading, i.e., synchronization; or agents separate into several subgroups with different headings and never merge again especially when the space is unlimited (see Fig. 1(a)–(b)). Papers [27] [28] show that when the density of the group is high enough, the group will self-organize to synchronization with large probability. It is more likely to separate into several sub-groups than in model with periodical boundary space. So an important question is: how do we intervene in the system and lead all agents synchronizing towards a desired heading if the self-organized heading is not what we expect or the group separates into subgroups? In this paper, one intelligent shill is added into the group to guide the system to synchronize towards a desired heading (see Fig. 1(c)–(d)). Because the shill is artificial, we can design its moving strategy for this purpose. The rest part of this paper solves the following problem:

#### Problem definition

considering the Viksek-like model defined above. Given any initial configurations that consist of 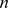 normal agents randomly and uniformly distributed inside a finite size area (a square with length 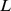) and with headings randomly picked up from 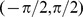 with uniform probability. Agents move in the unlimited two-dimensional space following local rules of (2) and (3). A shill is added to guide all normal agents synchronizing towards a desired heading 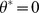, i.e., 
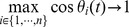
 when 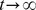. What is the strategy (dynamics) for the shill to complete the task?

As we have pointed out, the difficulty is how the shill decides the moving direction for each step using online feedback information. Especially, how a shill moves from one target agent location to another without putting negative effects on others is challenging. This paper gives a solution by proposing a novel strategy for the shill called ‘consistent moving’ (demos see Video S1). In the following, attributes of the shill are described, and then the moving strategy is introduced.

The position, heading and speed at time 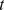 of the shill are denoted as 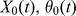 and 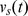 respectively. The neighborhood structure of a shill is the same as normal agents. The shill will affect a normal agent 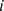 if it is in the neighborhood of agent 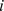, i.e., 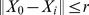. Since normal agents treat a shill as a normal one, normal agents still keep their update rules defined in equations (2) and (3) except that the definition of neighborhood 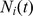 includes the shill as well:
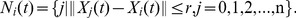
(4)


Therefore, the way normal agents treat a shill is the same as the way they treat normal ones. So shills have only the same strength of influence on neighbors as normal agents do.

There are differences between the shill and the normal agent: (a)The shill does not need to follow the normal agent's local rule of equation (2). Its moving strategy can be re-designed, which is called ‘consistent moving’ in this paper; (b)The shill can use online feedback information of normal agent location; (c)The shill is allowed to speedup and slowdown during the evolution, so 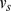 is not a constant but satisfies 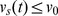 for all 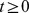, where 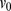 denotes the *maximal shill speed*.

**Figure 2:**
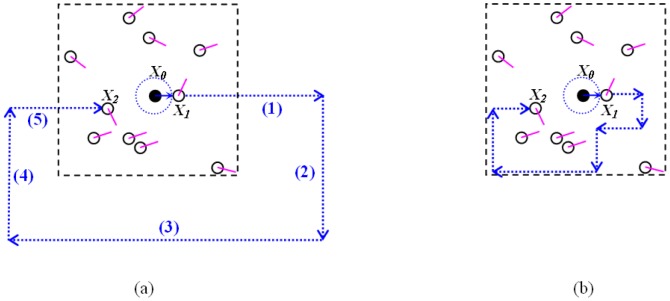
Examples of the moving route from location of agent 1 (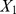) to location of agent 2 (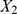) for the shill (starting from location 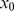). The big dash-line square indicates the current group area (note that to show ideas of the shill route, for convenience, the group area shown here is supposed to be static. In fact, the actual consistent moving strategy considers the cases that normal agents are moving when the shill moves, i.e., the group area keeps changing, which is much more complicated.). Two moving routes for the shill are shown: **(a) a simple U-turn route**: first it goes forward to a location which is much far away from the whole group(dash line part (1)), then it makes a big U-turn (dash line parts of (2)–(3)–(4)) far away outside the group area and gets back to the left side of the whole group, finally goes forward and affects agent 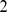 (dash line part (5)). Its heading is set to be zero in parts (1) and (5), while it can have different headings during (2)–(3)–(4) because there are no neighboring agents. **(b) A more efficient route** for the shill moving from agent 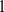 to agent 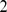. Radius of the small dash line circle centered at the shill represents the neighborhood size 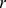 which is the same as the radius of normal agents. The shill tries to find a shorter route which maintains a ‘safe’ distance (larger than 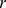) away from any normal agent when its heading is not zero. It is much more efficient than the simple U-turn route.

To affect the heading of a normal agent 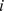, the shill should be inside the neighborhood of agent 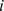. While the shill is neighboring to agent 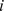, if the heading of the shill 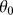 is zero (the desired heading), 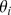 (the heading of agent 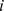) will be drawn towards to direction zero rapidly within a few steps, which is considered as a positive effect on agent 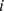; if 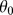 is not zero, the shill will lead 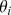 to other direction, which is considered as a negative effect on agent 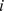. So to guide agent 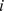 and make 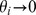, the shill first moves close to the left side of agent 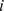 (see Fig. 2(a)), then changes to heading zero, moves forward and hits agent 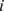. It should keep staying with agent 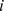 for a few steps till 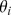 is small enough (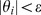). Therefore, to guide one agent is not difficult. But to guide the whole group we need to consider two basic problems in the shill moving strategy:

(1) When to affect a normal agent? Does the shill need to affect all normal agents one by one repeatedly? Which agent should be selected as the next target to be affected? What are the criteria for selection? Jadbabaie et al. [20] gave a preliminary result on a condition of synchronization for the linearized Vicsek's model, which showed that the connectivity of the union of neighbor graphs within some time-interval can guarantee synchronization. Inspired by this result, the first principle of the ‘consistent moving’ strategy is: ***the shill should periodically affects every agent directly or indirectly with heading zero***.

In a period, the order for target agent selection does not affect whether or not the shill can complete the task, but it might affect the convergence time. The simplest schedule in a period is to affect agents in a fixed order: 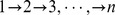. But we found from simulations that if an agent shows the trend of moving far away from the group centroid, it should be selected by the shill with a high priority. Otherwise that agent will move far away from the group and the shill will have to spend much more time to catch up with it. So in this paper the agent which has the biggest growth of distance from the group will be picked up first.

(2) What is the moving route of the shill from one location to the next target agent location? This is the core of the strategy. Note that if the shill is not always moving with the desired direction (heading zero), it will have negative effects on its neighboring normal agents. However, if the shill moves with a fixed heading of zero, it could never get to some locations. One solution is to allow the shill to change heading but make sure not to put negative effect on any normal agent during its movement. This can be described as the second principle: ‘***the shill should always keep heading zero (the desired heading) if it has neighboring agents; only when it has no neighbors can it have a non-zero heading***’. A subtle algorithm is designed based on this principle. It is a comprehensive algorithm for an intelligent shill.

The simplest idea for a shill to move from one target agent location to another is shown in Fig. 2(a). It first goes forward with heading zero till far away from the group, then makes a big U-turn outside the group area and gets back to the left side of the whole group, finally hits the target with heading zero. With a speed which is faster than 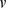 the shill can make this kind of route and it will not meet any normal agent during the U-turn process even considering the normal agents are moving. The mathematical proof shows that the shill with a limited 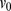 can affect every agent inside a period and lead the group to synchronization.

However, this simple route is not efficient. In fact, the shill can find a shorter route shown in Fig. 2(b). The shill can move inside the group area with a much more refined and careful path as long as it avoids meeting any normal agents when its heading is not zero. This shortcut is very subtle in this case. It is produced by a finite-state machine (See Appendix S1), which is based on the simple route idea with some heuristics for finding shortcuts.

With a shill, the overall system evolves in this way: firstly, 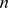 normal agents and a shill randomly and uniformly distributed inside a finite size area. During evolution, at each step, normal agents update their headings according to equations (2) and the shill updates its heading according to the finite-state machine (Fig. S1 in Appendix S1). And then they simultaneously update their positions using equation (3). In computer simulations, the evolution stops when the system reaches the synchronization criterion, i.e., 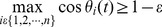 with 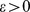.

The section of theoretical result below will give a rough upper-bound for the speed of the shill which can guarantee the evolution will stop in finite steps for any 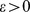. This means that the shill with a speed not larger than the bound value can guide all agents' headings converge to zero. The computer simulation results will exhibit that with 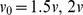 and 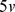, the evolution can stop in finite steps for 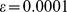, which means that in practice the shill can synchronize the group with a speed much lower than the theoretical bound.

## Results and Discussion

### Theoretical result

For compact construction and easy understanding, we will present the theoretical result in this subsection, and give a detailed mathematical proof in Appendix S2.

From the above section, we know the following two principles, denoted by 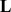, are satisfied:

- **The shill always keeps the desired heading** 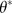 **when it has neighboring agents. That is to say, the heading of the shill felt by normal agents is always** 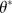 **;**
- **All agents will be directly or indirectly affected periodically by the shill in a time period** 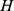 **.**

Note that in (i), 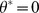 in this paper. But in fact, the proof is truth for any 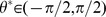.

An essential problem we concerned about is that can the shill with a limited speed accomplish periodical intervention? We know that if all normal agents can be covered by a limited circle in every time step, the shill can finish its task with a limited speed. On the other hand, if the minimal circle which covers all normal agents becomes bigger and bigger during evolution, the shill will have to keep accelerating to make sure it can catch up and affect all agents in a fixed period. Thus, the question can be translated to whether all normal agents can be covered by a limited circle during the whole evolution process.

**Figure 3:**
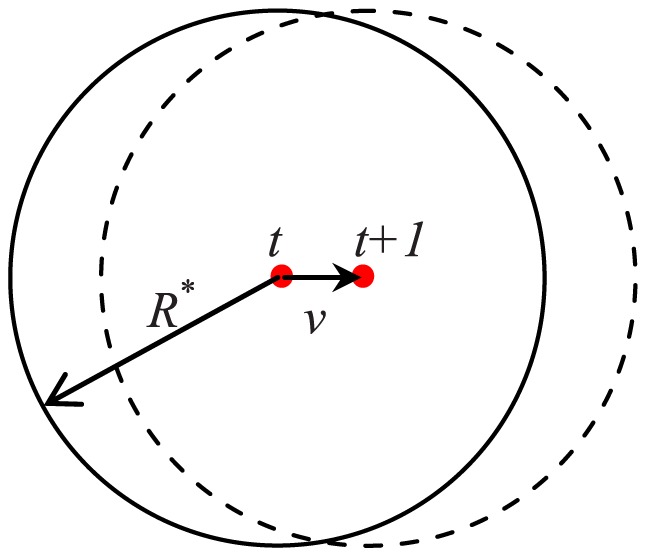
The locations of all normal agents can be covered by a circle centered at a reference point (little dot) with a fixed radius 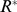 at every step. Solid-line circle represents the location area of time 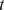. As the reference point is moving with speed 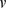 and desired direction 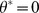, the location area of time 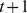 is presented by the dashed-line circle.

For the group, we define a dynamic reference point 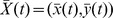 as follows:
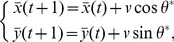
(5)where 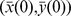 is the center of the minimal circle covering all normal agents in the initialization. Fig. 3 shows the dynamical circle covering locations of all normal agents. Note that the reference point is virtual and has no influence on the other agents. We will see that at time 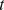 all normal agents will be covered by a circle centered at 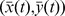 with a fixed radius.

**Theorem 1** *If the shill strategy satisfies condition* 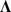 *, it can lead*

- *headings of all normal agents exponentially converge to the desired value* 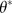 *, and*

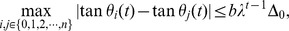

*where*

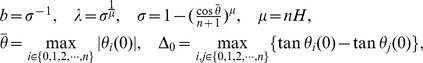

- *locations of all normal agents can be covered by a circle with a fixed radius* 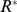 *at every step, and*

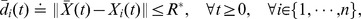

*where*

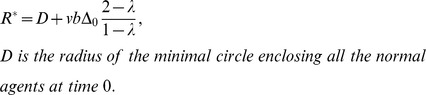

The mathematical result shows that with the help of a shill using ‘consistent moving’ strategy, the size of the group area is always limited during the evolution. This means the distance between any two normal agents is always limited. So with a limited speed, the shill can move from one agent's location to any other agent's location within limited steps.

It can be found that with some speed not larger than 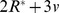, the shill can pass across the whole group in one step and have no neighbors in the next step. Thus, for any given period 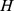, we can set 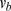 – the bound value of the shill speed, to be 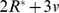. In particular, some speed not larger than 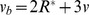 can ensure the shill to take the big U-turn route shown in Fig. 2(a) and directly affect each normal agent at least once in a period of 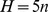. Therefore, the shill can accomplish its task to synchronize all normal agents with a limit speed.

Note that 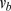 is only a rough upper-bound value, which is in order to show that with a limited speed the shill can perform the periodical intervention and synchronize the system. Actually we can see in the following computer simulations, the shill with a speed much lower than 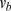 can synchronize the group in practice.

### Computer simulations

The settings of parameters in simulations are as follow: neighborhood radius 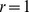 and normal agent speed 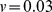, which are adopted from the Vicsek's paper [19]; the initial location area is a square with length 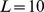; different group sizes 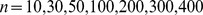 are tested, so the corresponding initial densities 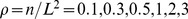; different shill maximal speeds 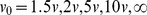 are tested. 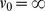 means that once the shill affects the target agent, it will slow down a bit if there are neighbors around, otherwise it will get to the position of the next target agent in the next step, no matter how big the distance between them. For each realization, the simulation will stop when the synchronization criterion 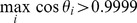 is satisfied. For each 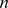 and 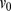, we test 500 realizations starting from different random initial configurations. For each realization, the total number of steps (denoted as *totalStep*) is measured, which means that the system is synchronized after *totalStep* steps.

#### Can one intelligent shill synchronize the system within limited steps?

**Figure 4:**
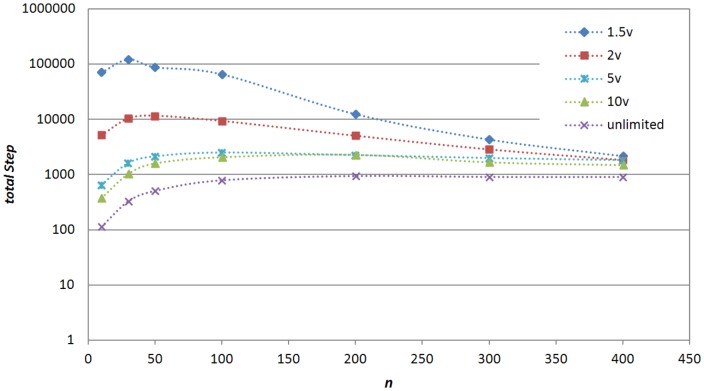
Mean totalStep (over 500 realizations) for synchronization (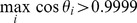) under different settings of 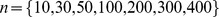 and 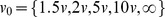, when one intelligent shill is added.

How does the shill maximal speed 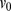 affect the performance? What if 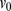 is much smaller than the theoretical bound 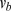
? How does the group size 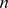 affect the performance? Fig. 4 answers the above questions. It shows that one intelligent shill with ‘consistent moving’ strategy can synchronize the group within limited steps.

With 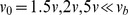, the shill still works for the synchronization task within finite steps. Because small 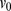 requires longer time for the shill to catch up with target agents, the system needs a larger *totalStep* to reach synchronization. Note that 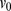 should be larger than 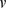 (the speed of normal agents), otherwise the shill will never catch up with and affect a running-away normal agent.

The impact of group size 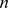 is twofold. When the group size 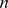 is larger, the shill needs to affect more agents. From this aspect, larger 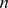 requires more steps for synchronization. On the other hand, because the initial area is fixed, larger 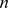 means higher initial density. As the theoretical result shows, higher density implies larger probability of self-organized synchronization [26]. Therefore, larger 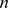 (density) means that the system is more likely to self-organize to synchronization and the group is more connected. The radius of the minimal circle which covers all normal agents will be smaller. It takes less time for the shill to guide the group. From this aspect, larger group needs less intervention from the shill. These two effects work together and the overall impact of 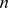 can be found from Fig. 4: when 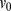 is small, cases with small 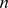 (low density groups) take a longer time (larger totalStep) for convergence; when 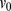 is large, cases with larger 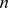 take a longer time for convergence. For example, the peak of *totalStep* for the case of 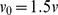 locates at 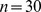, and then shift to 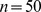 for 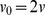, 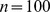 for 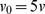 and 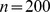 for 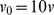. This is because when the shill moves with a slow 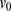, it takes a long time to catch up with the target agent, so the group is more likely to separate into several subgroups soon in the beginning if the initial group density is low. Consequently distances between two target agents become very large and the shill takes a much longer time to move from one target agent location to the next target agent location. Thus, the convergence time is significantly increased. When 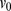 increases, the shill can efficiently stop the separation in the beginning and quickly move to the next target agent location, so what matters the *totalStep* is the number of normal agents that are needed to be affected in one period. Therefore, cases with larger 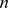 need longer time for convergence.

It is worthy to point out that small 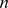 (low initial density) is more challenging for the shill when 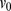 is small. Because for dense groups, the best choice of the shill is the U-turn route which can be produced by simple algorithms. But for low density cases, the group tends to separate into several subgroups more quickly in the beginning; to be efficient, the shill needs to find subtle shortcuts passing inside the group like Fig. 2(b), which can significantly reduce the convergence time compared to the simple U-turn route. Our method, the finite-state machine shown in Fig. S1 of Appendix S1, can automatically produce both simple U-turn route for low density groups and subtle shortcuts for dense groups. So the shill using this strategy shows computational intelligence and it can efficiently guide the group.

#### Comparing with a number of fixed-heading shills - model without noise

As mentioned in the introduction section, adding a number of fixed-heading simple shills is a method to coordinate headings of a group with dense population. It is proved that if the proportion of fixed-heading shills (informed agents) is equal to or greater than 
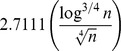
 [30], all agents can be led to move along the expected direction with probability of 1 when the group initial density is big enough. Compared to the case of adding one intelligent shill, which method is better? What if the group density is low? We know that the group can converge to heading zero with one intelligent shill, so do headings of all agents converge to zero with some fixed-heading simple shills? If not, what is the convergence value?

**Figure 5:**
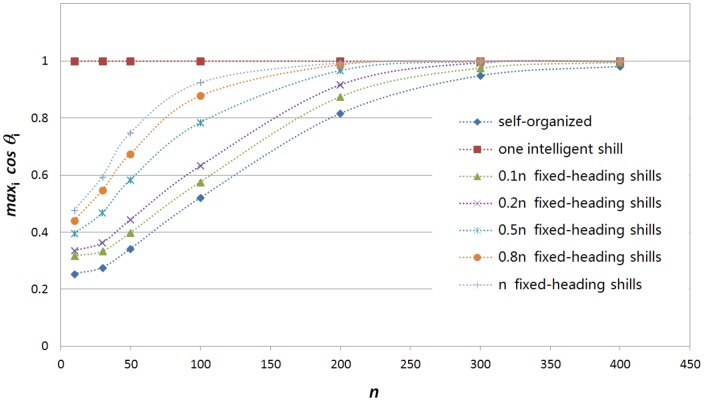
Synchronization level(mean 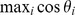) for 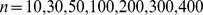 respectively in the following cases: self-organized without any intervention; one intelligent shill with 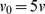 is added into the group; 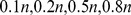 and 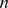 fixed-heading simple shills are added into the group respectively.

In simulations, 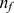 simple shills with fixed heading zero are added into the system. They are randomly distributed uniformly in the initial square area. And then let the system evolve till 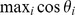 converges to a static value. 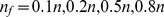 and 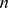 are tested for 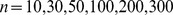 and 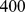 respectively. Fig. 5 shows the *synchronization level*, i.e., mean convergence value of 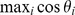, for three cases: ‘adding 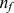 fixed-heading shills’, ‘self-organized’ (without intervention) and ‘adding one intelligent shill’. We can see that for the self-organized behavior, the *synchronization level* is the lowest, but it increases with 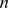. It almost self-synchronizes when 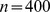. This is the reason why we do not test 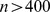 for 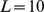. For the case of adding one intelligent shill, the convergence value is always 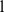. For the case of adding some fixed-heading shills, larger 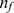 leads to higher *synchronization level*. But its level is low when 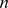 is small. For example, in the system consists of 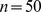 normal agents and 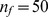 fixed-heading shills, the *synchronization level* 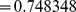. So for low density groups, one intelligent shill performs much better than a number of fixed-heading shills when measured by the *synchronization level*. This is because low density groups need much more fixed-heading shills to directly or indirectly influence all normal agents. But an intelligent shill with feedback information can periodically affect every agent and synchronize the group.

#### Comparing with a number of fixed-heading shills - model with noise

In the original Vicsek' model [19], the heading update rule includes a noise part: 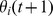 equals to the angle of the sum of the velocity vectors of neighbors plus a random number 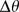 chosen with a uniform probability from the interval 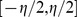. They define 
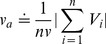
 as the order parameter, which can be regarded as the *synchronization level*. 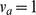 if headings of all agents are the same, i.e., the system synchronizes. In this case with noise, does the intelligent shill work for the model with noise? How does it compare to the method of adding some fixed-heading shills? What are the performance characteristics of both methods?

**Figure 6:**
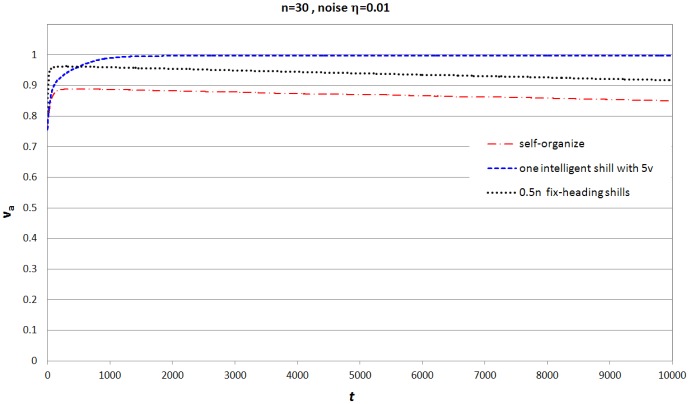
Mean value (over 500 realizations) of 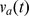 during time evolution for 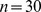 and 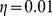 under 3 scenarios: (1)self-organized; (2)one intelligent agent with 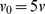 is added into the group; (3)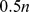 ( = 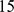) fixed-heading shills are added into the group.

Fig. 6 shows that with small noise, how mean value of *synchronization level* changes during time evolution (from 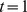 to 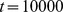) for both methods. The group *synchronization level* is decreasing obviously during time evolution even with the help by a number of fixed-heading shills; while adding one intelligent shill can maintain a high *synchronization level*. This is because the method of adding fixed-heading shills has a serious problem when facing noise: once an agent moves away (driven by noise) from the shills' neighborhood, it might never be neighbors of shills since the space is unlimited and shills are moving with a fixed direction. Therefore, fixed-heading shills will lose more and more neighbors, and have less and less impact on the group during evolution. On the other hand, a theoretical result [29] shows that if the shill can affect all agents periodically, headings of all agents fluctuate around the desired heading rather than reaching it, and the range of the fluctuation depends on the magnitude of the noise. This indicates that it is impossible to maintain fully synchronization (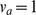) in the model with noise, but if the shill can periodically affect all agents, it can maintain a reasonable *synchronization level*. With feedback information, the intelligent shill can adjust its moving direction and try to catch up with those running-away normal agents instead of losing them.

**Figure 7:**
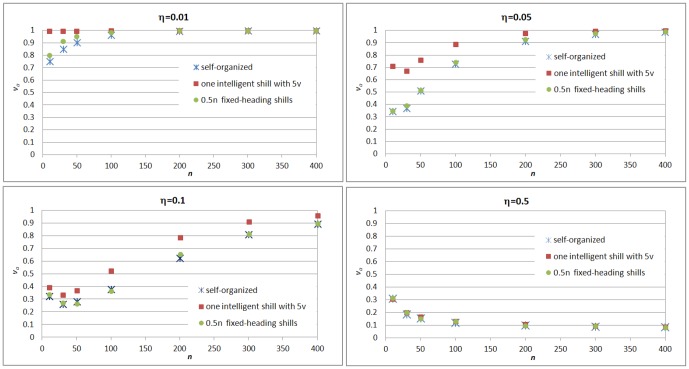
Mean value (over 500 realizations) of 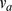 at 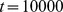 with noise 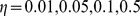 respectively. Three scenarios are considered for each case: (1)self-organized; (2)one intelligent agent with 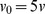 is added into the group; (3)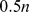 fixed-heading shills are added into the group.

Fig. 7 demonstrates the mean 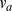 at 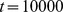 for both methods under different settings of noise 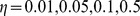 and group size 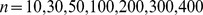 respectively. It shows that for small noise 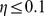, adding one intelligent shill (with 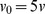) can achieve a high *synchronization level* which is obviously higher than adding 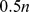 fixed-heading shills, especially for low density groups. For 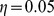 and 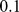, compared to the case of ‘self-organized’, adding one intelligent shill can significantly promote the *synchronization level*, whereas adding 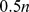 fixed-heading shills can not. But for the case with big noise 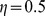, none of them can promote the *synchronization level*. This is because with fluctuation the distance between agents might keep increasing and become too large; then the shill can not be able to periodically affect all agents with a limited speed.

This result shows that for small noise, one intelligent shill using ‘consistent moving’ strategy can significantly promote synchronization and perform much better than the method of adding some fixed-heading shills, because the intelligent shill can use online information to adjust its heading and try to keep up with every agent. Therefore, online feedback information of agent location is important in this sense, especially in the procedure of next target agent selection. This shows the advantages of intelligence through feedback mechanism. We notice that tightly connected group(group with high density) has low tendency of dispersion caused by noise because each agent in the group has many neighbors that can cancel out most of the noise effect. It suggests future study of the intelligent shill to achieve better synchronization level in the noise model: for example, first the shill tries to drive all normal agents moving towards a center to form a tightly connected group, and then the shill use the ‘consistent moving’ strategy to guide synchronization.

In a word, the most important difference between methods of adding a number of fixed-heading shills and adding one intelligent shill is the feedback mechanism which brings big benefits but requires observation of information (in fact, global information is not necessary during the procedure of shill moving from one location to the next target agent location, see Appendix S1). Except this, other difference lies in: (1)the former is a method that works in the probabilistic scenario for high density groups, while the latter is a deterministic method for any initial configuration for any density. The former might not lead the group converge to the desired heading starting from some configurations, especially when the group density is low. (2)The former needs a number of shills, while the latter needs one; (3) Shills in the former method move with the same speed as normal agents, whereas the shill in the latter can adjust its heading based on feedback information and it needs a speed which must be faster than normal agents; (4)As considering the model with noise, computer simulations show that the latter performs much better than the former because the power of feedback.

## Conclusions

We have studied nondestructive intervention by adding one intelligent shill and proposed a smart strategy for the intelligent shill to coordinate synchronization of a Vicsek-like model using online feedback information, which is the first complete and feasible algorithm for this purpose. The strategy obeys two principles: (1)the shill should directly or indirectly affect all normal agents periodically; (2)by using online feedback information of normal agents, the shill should avoid putting negative effects on normal agents when it is moving in a non-desired direction. According to these two principles, we have designed a finite-state machine for the dynamics (moving algorithm) of the shill which can produce refined shortcut route based on the simple U-turn route. This is called ‘consistent moving’ strategy. The mathematical analysis gives a bound 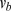 which can ensure that the shill using ‘consistent moving’ strategy with a speed not larger than 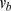 can affect all normal agents inside one period. Therefore, the group can converge to the desired heading. This means that an intelligent agent with ‘consistent moving’ can synchronize the group with a limited speed. Computer simulations show that a shill with speeds 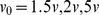 which are much lower than 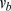 can lead the group to a synchronized state (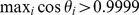) within finite steps. With larger 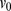, the group can converge faster. Compared to the method of adding a number of fixed-heading shills, our method performs better in terms of *synchronization level*, especially for low density groups and model with noise. This is mainly due to intelligence of the shill with feedback mechanism.

Based on our approach, there are a number of possible future studies: (1) The goal of this paper is to prove that one intelligent shill can guide the group to synchronization and there exists such a strategy for the shill. We believe that the efficiency of the algorithm for the shill can be improved by changing the heuristics and some parameter settings. (2) In this paper, the desired heading is zero. Actually, theorem 1 is true for any desired heading 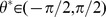. The related ‘consistent moving’ strategy can be obtained by the state transition diagram (Fig. S1 in Appendix S1) with some minor modifications. (3) The ‘consistent moving’ strategy provides an algorithm of how a shill moves from one agent location to another without putting any negative effect on the group. This provide a base for further development of more efficient strategies for the shill, as well as strategies for other coordination purposes of Vicsek-like models, such as circus movement of group, tracking of desired route, obstacle avoidance, etc. Moreover, the strategy of the shill to lead synchronization can be a base for other coordination purpose, because it will be much easier to guide the group to turn or move to a destination after the system is synchronized; (4)The strategy for the shill in 3-D space model, and the strategy for the shill in a speed consensus problem of a continuous model that includes both magnitude and direction deserve further investigation; (5)To adding more than one intelligent shills into the group, and to design strategies for the intelligent shills to cooperate or compete for collective motions of the group will be a challenging topic.

In fact, the intention of this paper is not only to complete a special coordination task of a specific MAS, but also to suggest a nondestructive intervention method by adding intelligent shills for other MASs as well. For example, adding intelligent shills in a multi-player group to change the group decision, adding intelligent shills in crowd to avoid panic, adding intelligent shills on internet to intervene public opinion, etc. On the other hand, this idea also provides a possible solution for the design of man-made MASs. To design a self-organized MAS to achieve an expected collective behavior requires a lot of skills in the design of local interaction rules. Adding one or more intelligent shills to coordinate the collective behavior can release designers from the hard work of subtle design.

In one word, this approach demonstrates the importance of using feedback for intervention. Intelligent shills can significantly outperform simple fixed-heading agents. As more attention has been paid to control and intervention of collective behavior, we need to design intelligent strategies for effective and efficient intervention for complex multi-agent systems and varieties of intervention purposes, not limited to synchronization only, but other patterns of collective movements as well.

## Supporting Information

Appendix S1 **Algorithm of ‘Consistent Moving’.**
(PDF)

Appendix S2 **Mathematical Analysis.**
(PDF)

Video S1 **Video to show the idea of how one intelligent shill intervene into the group and lead synchronization.**
(WMV)
