# Supplementary material for: Nondestructive Intervention to Multi-Agent Systems through an Intelligent Agent
Source: PLoS One. 2013 May 2;8(5):e61542. doi: 10.1371/journal.pone.0061542 (PMC3642172; doi:10.1371/journal.pone.0061542)
Supplement: Appendix S1 — Algorithm of ‘Consistent Moving’. (PDF) [file pone.0061542.s001.pdf]

Supporting Information

Appendix S1 - Algorithm of 'Consistent Moving'

The algorithm of the strategy of the shill is described by a finite-state machine, which shown as a state transition diagram in Fig.S1. According to this diagram the shill will move inside the group without putting negative effect on normal agents, because it will always take heading zero when it has neighbors. The simple U-turn route is a special case that can also be produced by this state transition diagram, if the shill speed is very large. Otherwise with a low and median speed, its moving route will be much more subtle like the ones shown in Fig.2(b).

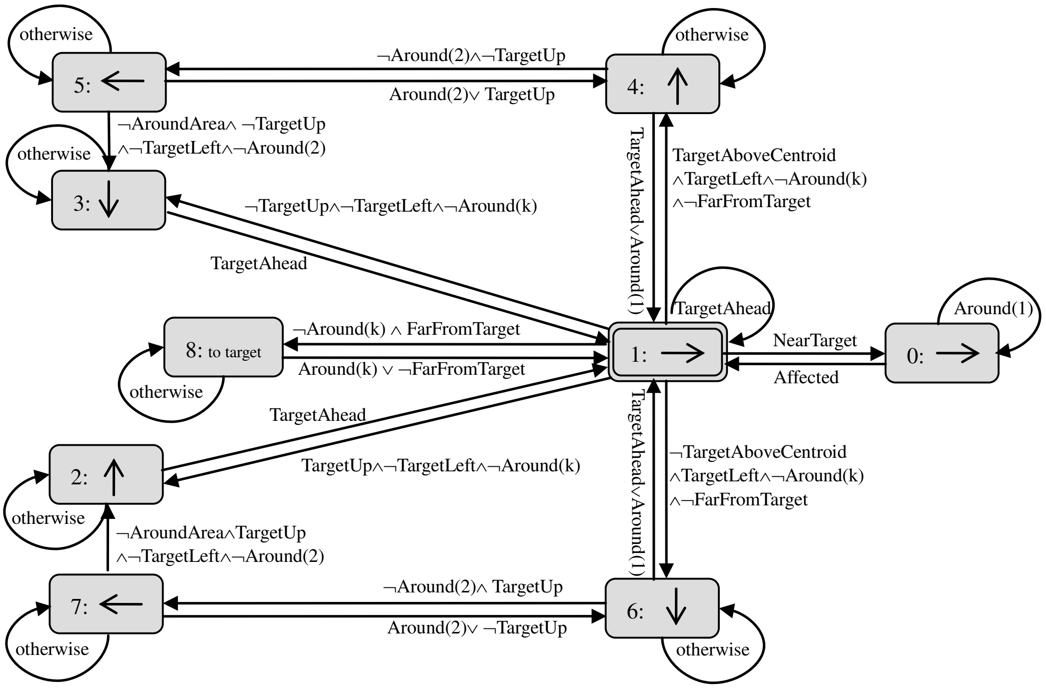

Figure S1: State transition diagram of how the shill decides its moving direction in 'consistent moving'. The heading of the shill in each state is indicated by the arrow inside the state box. For example, heading of state 0 and state 1 are zero, while headings of state 3 and state 6 are $-\pi/2$. In particular, heading of state 8 is pointing to the current location of the target. State 1 is the beginning and ending state of a loop. Once a target agent is affected, the new loop begins for the next target agent. Loop of state $1 \leftrightarrow 6 \leftrightarrow 7 \rightarrow 2 \leftrightarrow 1 \leftrightarrow 0$ is for the route bypassing from underneath. Loop of state $1 \leftrightarrow 4 \leftrightarrow 5 \rightarrow 3 \leftrightarrow 1 \leftrightarrow 0$ is for the route bypassing from above.

Explanations of Boolean functions for each transition of Fig.S1 are listed below:

Affected: it returns true after the target is affected by the shill.

TargetAhead: it returns true if keeps moving in heading zero the shill will hit the target agent.

Around(k): it returns true if during the next k steps, the shill might have one or more neighboring agents (i.e., the shill might affect one or some normal agents).

TargetAboveCentroid: it returns true if $target.y > avg.y$, where $target.y$ is the $y-$coordinate of the target agent, and $avg.y = (\sum_{i=1}^{n} y_i)/n$ is the $y-$coordinate of the centroid of n normal agents.

TargetUp: it returns true if the location of the target agent is above the shill, i.e., $target.y > shill.y$.

TargetLeft: it returns true if the target agent locates in the shill's left side, i.e., $target.x < shill.x$.

AroundArea: it returns true if it will not have any neighboring normal agent during its movements upwards/downwards to the horizontal target position ($target.y$).

NearTarget: it returns true if the shill is neighboring to the target.

FarFromTarget: it returns true if the distance between the shill and the target is bigger than $G * r$, where G is a heuristic value($G = 5$ in the simulations).

otherwise: This is not a specified condition function. It is true if conditions on other transitions are false. For example, if from state 8, it does not satisfy conditions to transit to state 1, it will keep state 8.

For example, at time t, if the shill is in state 8, its heading is pointing to the current location of target agent at time t. It checks the conditions: if *Around(k)* is $false$ and *FarFromTarget* is $true$, at time $t + 1$ it will change to state 1 and its heading is updated to be zero. Otherwise, it will keep in state 8 at time $t + 1$ with its heading pointing to the location of target agent at time $t + 1$.

The time computational complexity of all above Boolean functions are not high: $O(1)$ for *Affected*, *TargetAhead*, *TargetAboveCentroid*, *TargetUp*, *TargetLeft*, *NearTarget* and *FarFromTarget*. To reduce the computation, *Around(k)* and *AroundArea* are considered in the worst case, since exact prediction is not required in these heuristic functions. For example, when *Around(k)* is checked, it is not necessary to simulate k steps evolution of the whole system to see whether during k steps the shill will have any neighboring agent. Instead, we check whether there are agents inside the circle of radius kr centered at the shill. So the computational complexity for *Around(k)* and *AroundArea* are $O(n)$.

The current strategy needs global information which is demanding. However, as we can see from the case of adding simple fixed-heading shills that do not require global information, the system will fail in synchronization starting from some configurations. So in this sense global information is necessary. But it does not mean that we need to know location information of all normal agents at each state. Actually, only local information is needed to compute Boolean functions of *Affected*, *Around(k)* and *NearTarget*. While *TargetAhead*, *TargetUp*, *TargetLeft* and *FarFromTarget* need no information of normal agents but only the location information of the target agent. *AroundArea* needs local sense of locations of near agents and the location information of the target agent as well. The only one that needs location information of all agents are *TargetAboveCentroid* . Actually, this function is not necessary. Indeed, the shill can always take the loop from underneath or above (see Fig.S1), the only problem is that it might be less efficient than using the centroid information to decide going underneath or above. As another solution, we might try to find some way of determining the approximate centroid without complete knowledge of the normal agent positions.

The following Pseudo-code roughly shows the idea of the complete algorithm for the system evolution with a shill. After initialization of n normal agents and the shill, the system evolves until it is synchronized, i.e., $\max_{i \in \{1,2,\cdots,n\}} \cos\theta_i(t) \geq 1 - \epsilon$ with $\epsilon > 0$. During evolution, normal agents update their headings and positions according to equations (2) and (3). Meanwhile, the shill is moving according to the 'consistent moving' strategy. Once a target agent is affected, the function of *target_agent_selection* will return the next target agent from the *unaffected-set* according to the heuristic mentioned above and a new period begins if all agents are affected. The program will stop when the maximal cosine value for all headings is larger than $1 - \epsilon$.

Procedure *main*

0. t=0;

initialization:

$X_i(\theta)$ = randomly and uniformly picked up from a square of length L, for $i = 0, 1, ..., n$.

$\theta_i(0)$ = randomly and uniformly picked up from $(-\pi/2, \pi/2)$, for $i = 1..n$.

$state = 0$; *//this refers to the state number in the state transition diagram*

$\theta_0(0) = 0$; *//heading of the shill*

$j = target\_agent\_selection$;

1. **while** $\max_i \cos\theta_i(t) > 1 - \epsilon$ **do** *// check whether the group is synchronized*
2. $t = t + 1$;
3. **if** $j = 0$ **then** $j = target\_agent\_selection$;
4. updates $\theta_0(t)$ and *state* according to the state transition diagram (fig.S1)
   if *state= 0* **then** $j = 0$. *//if the targer agent is been affected, it will find another target*
5. updates headings and positions of n agents according to equations (2) and (3).
6. updates position $X_0(t)$ of the shill according to equation (3).
7. **end while**.

Note that if we also want to make sure the shill can periodically affect all agents in a given period of H, the shill can still try to use small v_0 in most of time as possible as it can, except when there are n' agents unaffected and there are no more than $5n'$ steps left in the period. In this situation, the shill should speed up to a big value (which is not larger than v_b). Then it can affect one unaffected agent using 5 steps (it is proved in Appendix S2 that the shill can move from one agent position to another using the big U-turn route shown in Fig.2(a)) with a speed which is not larger than v_b. Thus, it can accomplish to affect n' unaffected agents in the remained time $5n'$ steps. Then we can make sure all agents are affected in one period of H steps.
