# Supplementary material for: Nondestructive Intervention to Multi-Agent Systems through an Intelligent Agent
Source: PLoS One. 2013 May 2;8(5):e61542. doi: 10.1371/journal.pone.0061542 (PMC3642172; doi:10.1371/journal.pone.0061542)
Supplement: Appendix S2 — Mathematical Analysis. (PDF) [file pone.0061542.s002.pdf]

Supporting Information

Appendix 2 - Mathematical Analysis

First, we use *directed* graph $D_t = (V, E_t)$ to model the interaction topology among agents at time t. Here, the vertex set $V = \{0, 1, 2, \cdots, n\}$ consist of a shill (denoted by agent 0) and n normal agents. $E_t \subset V \times V$ is the arc set, and an arc $(i, j) \in E_t$ means that agent j can receive information from agent i at time t, i.e., i is a neighbor of agent j. D_t is called neighbor graph.

For a directed graph (digraph) D, a *path* from vertex i to j is a sequence of distinct vertexes $i_0, i_1, i_2, \cdots i_m$, where $i_0 = i, i_m = j$ and arc $(i_l, i_{l+1}) \in E, 0 \leq l \leq m-1$. A digraph is called *strongly connected* if for any ordered pair of distinct vertices, there is a path in D connecting them. A digraph is said to have a *spanning tree* if and only if there exists a vertex $i \in V$, called *root*, such that there is a path from i to any other vertex. The *union* of a collection of digraphs $\{D_1, D_2, \cdots, D_k\}$ with the same vertex set V, is a digraph D with vertex set V and arc set $E = E_1 \bigcup E_2 \cdots \bigcup E_k$.

By using the above terminologies, the second condition of $\mathbf{\Lambda}$ means that the union of interaction graphs $\{D_{kH+1}, \cdots, D_{(k+1)H}\}$ has a spanning tree rooted at agent 0 for any $k \geq 0$.

Now, we will analyze the dynamic of all agents. For convenience, we introduce a transformation $\tan\theta_k(t)$, then for agent $1 \leq k \leq n$, the heading rule (2) can be rewritten as

$$\tan\theta_k(t+1) = \sum_{i \in N_k(t)} \frac{\cos\theta_i(t)}{\sum\limits_{j \in N_k(t)} \cos\theta_j(t)} \tan\theta_i(t), \tag{6}$$

where $N_k(t)$ contains the shill if it is in agent k's neighborhood.

For the shill, its heading updates complicatedly according to the state transition diagram. So it's difficult to describe its dynamic process by an equation. However, our goal is to synchronize the normal agents, and we can just focus on their headings. From the normal agents' view, the shill always keeps the desired heading θ^* because of i) of $\mathbf{\Lambda}$. Thus, the normal agents can assume

$$\theta_0(t) = \theta^*, \quad \forall t \geq 0.$$

Let $\tan\theta(t) = [\tan\theta_0(t), \tan\theta_1(t), \cdots, \tan\theta_n(t)]^\tau$. From the normal agents' view, the dynamic of the whole group can be written as the following matrix form:

$$\tan\theta(t+1) = P(t)\tan\theta(t), \tag{7}$$

where

$$P(t) \doteq \begin{bmatrix} 1 & 0 \\ \gamma(t) & A(t) \end{bmatrix}, \tag{8}$$

$\gamma(t) = [\gamma_1(t), \cdots, \gamma_n(t))]^\tau$ with

$$\gamma_i(t) = \begin{cases} \dfrac{\cos(\theta_0(t))}{\sum\limits_{k \in N_i(t)} \cos(\theta_k(t))}, & \text{if the shill is a neighbor of agent } i \text{ at time } t; \\ 0, & \text{otherwise.} \end{cases}$$

$A(t) = [a_{ij}(t)]_{i,j \in \{1, \cdots, n\}}$ with

$$a_{ij} = \begin{cases} \dfrac{\cos\theta_j(t)}{\sum\limits_{k \in N_i(t)} \cos\theta_k(t)}, & \text{if agent } j \text{ is a neighbor of agent } i \text{ at time } t; \\ 0, & \text{otherwise.} \end{cases}$$

From the problem definition, we know the initial headings of all agents belong to $(-\pi/2, \pi/2)$. Thus all elements of $P(0)$ are non-negative. It's also easy to check that the row sum of $P(t)$ is 1 independent of t. Recursively, we can get for any agent $i \in \{0, 1, \cdots, n\}$,

$$\min_{j\in\{0,1,\cdots,n\}} \tan\theta_j(t) \le \tan\theta_i(t+1) \le \max_{j\in\{0,1,\cdots,n\}} \tan\theta_j(t), \quad \forall t \ge 0. \tag{9}$$

Furthermore, from the monotonicity of function $\tan(\cdot)$, we know that the maximal heading of all agents is non-increasing, and the minimal heading is non-decreasing over time. So, at any time, the headings of agents belong to $(-\pi/2, \pi/2)$ and the elements of $P(t)$ are non-negative. Thus $\{P(t), t \ge 0\}$ is a sequence of stochastic matrix[3](cf. [3]).

From (7), we know

$$\tan\theta(t+1) = P(t)\cdots P(0)\tan\theta(0), \quad \forall t \ge 0. \tag{10}$$

Thus, in order to prove the synchronization, we need to study the product of stochastic matrices. Here, synchronization means the difference of headings of all agents are zero. Because the shill always affects normal agents with heading θ^*, the synchronization of system (7) means that headings of all agents converge to θ^*.

From (10), we know that if the product $P(t)\cdots P(0)$ tends to a matrix with the same row as $t \to \infty$, the difference among entries of $\tan\theta(t+1)$ tends to zero. A characterization of consensus is defined for the stochastic matrix $B = [B_{ij}]_{(m)\times(m)}$:

$$\tau(B) = \frac{1}{2}\max_{i,j}\sum_{s=1}^{m}|B_{is} - B_{js}|. \tag{11}$$

From [2], we know that for any stochastic matrices $B(1)$ and $B(2)$,

$$\tau(B(1)B(2)) \le \tau(B(1))\tau(B(2)). \tag{12}$$

Furthermore, the function $\tau(\cdot)$ also has the following property.

Lemma 2 *[2] Let $y = [y_1, \cdots, y_n]^\tau \in \mathbf{R}^m$ be an arbitrary vector and denote $\Delta y = \max_{i,j}|y_i - y_j|$. For a stochastic matrix $B = [B_{ij}]_{m\times m}$, if $z = Py,\ z = [z_1, \cdots, z_m]^\tau$, then we have*

$$\Delta z \le \tau(P)\Delta y.$$

Note that, Δy represents the difference among the entries of vector y. By Lemma 2 and formula (10), if $\tau(P(t)\cdots\ P(0))$ tends to zero, then we have $\lim_{t\to\infty}\Delta\tan\theta(t) = 0$ means the system converge to synchronization. Define

$$\lambda(B) = \min_{i,j}\sum_{s=1}^{m}\min(B_{is}, B_{js}). \tag{13}$$

If $\lambda(B) > 0$, then B is called *scrambling matrix*(cf. [2]). It is also easy to check that

$$\tau(B) = 1 - \lambda(B).$$

Thus, if B is a scrambling matrix, then $\tau(B) < 1$.

[3]A matrix is called stochastic if its elements are all nonnegative and the sum of each row is 1.

Based on the second condition of $\mathbf{\Lambda}$, we will see that the product of matrices $\{P(t)\}$ becomes scrambling matrix periodically, which leads to the exponential decrease of $\tau(P(t)\cdots P(t))$. To account for this, we need to study the relationship between the product of stochastic matrices $\{P(t)\}$ and the union of neighbor graphs $\{D_t\}$. Before elaborating this, we introduce some concepts and well-known results.

For a matrix $B \in R^{m\times m}$, its associated directed graph, denoted by $\Gamma(B)$, is a digraph on m nodes $\{1, 2, \cdots, m\}$ such that there is a directed arc in $\Gamma(B)$ from i to j if and only if $b_{ji} \neq 0$ (cf. [3]). From the definition of $P(t)$, we know the digraph associated with the matrix $P(t)$ is the neighbor graph D_t.

The composition of a directed graph G_1 with a directed graph G_2 (cf. [1]), is the directed graph with vertex set $\{1, 2, \cdots, m\}$ and arc set defined as follows: (i, j) is an arc of the composition just in case there is a vertex k such that (i, k) is an arc of G_1 and (k, j) is an arc of G_2.

According to the concept of associated directed graph $\Gamma(B)$ for a matrix B, we know that for any two matrices B_1, B_2, the composition of $\Gamma(B_1)$ with $\Gamma(B_2)$ is in fact the digraph $\Gamma(B_2B_1)$.

A neighbor-shared graph is a graph that each pair of two distinct vertices share a common neighbor. From the definition of scrambling matrix, it is easy to see that the following two statements are equivalent: stochastic matrix B is scrambling; the associated graph $\Gamma(B)$ is neighbor-shared.

Based on the above concepts, we now introduce a result from which the periodic scrambling matrix hidden in the shill's strategy will be presented.

Lemma 3 *[1] For the rooted graphs with m vertices that each one has a self-arc, the composition of any set of $N \geq m-1$ such rooted graphs is neighbor-shared.*

From the second condition of $\mathbf{\Lambda}$, we know that the union of neighbor graph sequence $\{D_{kH+1}, \cdots, D_{(k+1)H}\}$ is a rooted graph for any $k \geq 0$. The union graph has $n+1$ vertices. Thus, the composition of n union graphs is neighbor-shared by Lemma 3. Combining the neighbor-shared graph with the scrambling matrix and defining $\mu = nH$, we know

$$\mathbf{P}((\mathbf{k}+\mathbf{1})\mu)\cdots\mathbf{P}(\mathbf{k}\mu+\mathbf{1}) \textbf{ is a scrambling matrix}, \ \forall \mathbf{k} \geq \mathbf{0}.$$

Thus, $\tau(P((k+1)\mu)\cdots P(k\mu+1)) < 1$, the exponential stable of $\Delta\tan\theta(t)$ becomes possible. The detailed analysis will be given in the proof of the theorem.

Up to now, we only investigate the headings of all agents. In fact, positions of agents affect the neighborhood which further affect their headings of the next step. And the headings will influence their future positions. That is, the headings and positions are coupled together. The reason why the above heading analysis can implement independently is that the motion of the shill is designed by us. We program the shill to affect every agent at least once in a fixed period. This guarantees the normal agents are affected by shill periodically, which further makes $\Delta\tan\theta(t)$ decrease. Combining with the above analysis, next we will give a complete proof of the main result.

Proof of Theorem 1

Let $\Delta_t \doteq \Delta\tan\theta(t) = \max\limits_{i,j\in\{0,1,2,\cdots,n\}} |\tan\theta_i(t) - \tan\theta_j(t)|$. Based on the previous analysis (9), we know that the function $\max\limits_i \tan\theta_i(t)$ is non-increasing, and the function $\min\limits_i \tan\theta_i(t)$ is non-decreasing. So, Δ_t is a monotonous sequence.

For the system (7), we define

$$\Phi(k+1, i) = P(k)\Phi(k, i), \quad \Phi(i, i) = I, \ \forall k \geq i \geq 0,$$

then,

$$\tan\theta(t+1) = \Phi(t+1, 0)\tan\theta(0), \tag{14}$$

and by applying Lemma 2, we have

$$\Delta_t \leq \tau(\Phi(t, 0))\Delta_0. \tag{15}$$

From the strategy designed for the shill, we know that for any $k \geq 0$

$$\Phi((k+1)\mu+1, k\mu+1) \text{ is a scrambling matrix.}$$

Let $\bar{\theta} = \max_{i\in\{0,1,2,\cdots,n\}} |\theta_i(0)|$. Since the maximal heading of all agents is non-increase and the minimal heading is non-decrease, for any $t \geq 0$, all the non-zero entries of $P(t)$ are large than or equal to $\dfrac{\cos\bar{\theta}}{n+1}$. Thus,

$$\lambda(\Phi((k+1)\mu+1, k\mu+1)) \geq (\frac{\cos\bar{\theta}}{n+1})^{\mu}, \quad \forall k \geq 0.$$

Hence,

$$\begin{array}{rcl}\tau(\Phi((k+1)\mu+1, k\mu+1)) & = & 1-\lambda(\Phi((k+1)\mu+1, k\mu+1)) \\ & \leq & 1-(\frac{\cos\bar{\theta}}{n+1})^{\mu} \doteq \sigma, \quad \forall k \geq 0.\end{array} \tag{16}$$

For any $t \geq 1$, there exists an integer $k_0 \geq 0$ such that $k_0\mu \leq t-1 < (k_0+1)\mu$. By (12), we have

$$\begin{array}{rcl}\tau(\Phi(t,0)) & \leq & \tau(\Phi(t, k_0\mu+1))\tau(\Phi(k_0\mu+1, (k_0-1)\mu+1))\cdots\Phi(\mu+1,1))\tau(P(0)) \\ & \leq & \sigma^{k_0} \leq \sigma^{\frac{t-1}{\mu}-1} = \sigma^{-1}(\sigma^{\frac{1}{\mu}})^{t-1}.\end{array}$$

Now, let $b \doteq \sigma^{-1}$, $\lambda \doteq \sigma^{\frac{1}{\mu}}$. Combining with (15), we have

$$\Delta_t \leq b\lambda^{t-1}\Delta_0, \tag{17}$$

which prove the first part of Theorem 1.

According to the update rules (3) and (5), with the fact that heading of the shill felt by normal agents is always θ^*, we know that at time t the distance between agent i, $i \in \{1,\cdots,n\}$ and the reference point $\bar{X}$ is

$$\begin{array}{rcl}\bar{d}_i(t+1) & \leq & \bar{d}_i(t) + 2v\left|\sin\dfrac{\theta_i(t)-\theta^*}{2}\right| \\ & \leq & \bar{d}_i(t) + v|\theta_i(t)-\theta^*| \\ & \leq & \bar{d}_i(t) + v\max\limits_{i,j\in\{0,1,2,\cdots,n\}}|\theta_i(t)-\theta_j(t)|.\end{array} \tag{18}$$

Because $\tan\theta - \theta$ is an increasing function when $\theta \in (-\pi/2, \pi/2)$, we have

$$\max_{i,j\in\{0,1,2,\cdots,n\}}|\theta_i(t)-\theta_j(t)| \leq \max_{i,j\in\{0,1,2,\cdots,n\}}|\tan\theta_i(t)-\tan\theta_j(t)|. \tag{19}$$

Thus

$$\bar{d}_i(t+1) \leq \bar{d}_i(t) + v\Delta_t. \tag{20}$$

Next, we will use mathematical induction to prove

$$\bar{d}_i(t) \quad \leq \quad R^*, \quad \forall t \geq 0, \quad \forall i \in \{1,\cdots,n\}. \tag{21}$$

It's obvious at $t = 0$. By using $\sigma < 1$ and the non-increase of Δ_t, it is easy to check that (21) is true at $t = 1$. Thus, the shill with a limited speed can finish its task at $t = 1$.

Now, Suppose (21) is true when $t \leq T$. Thus the shill can accomplish its task with a limited speed in time interval $[1, T]$.

Next, we will prove formula (21) is true at time $T+1$.

Case 1: if $T \leq \mu$

By using the monotonicity of Δ_t, we have for any $i \in \{1, \cdots, n\}$

$$\begin{aligned} \bar{d}_i(T+1) &\leq \bar{d}_i(0) + v\sum_{t=0}^{T}\Delta_t \leq \bar{d}_i(0) + v(T+1)\Delta_0 \\ &\leq \bar{d}_i(0) + v(\mu+1)\Delta_0 \leq R^*. \end{aligned} \tag{22}$$

Case 2: if $T > \mu$

By applying (15), we have

$$\begin{aligned} \bar{d}_i(T+1) &\leq \bar{d}_i(0) + v\sum_{t=0}^{T}\Delta_t \leq \bar{d}_i(0) + v\sum_{t=1}^{T} b\lambda^{t-1}\Delta_0 + v\Delta_0 \\ &\leq \bar{d}_i(0) + vb\Delta_0\frac{2-\lambda}{1-\lambda} \leq R^*. \end{aligned} \tag{23}$$

Combining the above two cases, we know that locations of all normal agents can be covered by a circle with radius R^* at time $T+1$. So, the shill with a limited speed can accomplish its task at time $T+1$. By using mathematical induction, we know (21) is true for all $t \geq 0$. So the speed of the shill can be bounded by a constant. ■

References

1. Cao, M., Morse, A.S., & Anderson, B.D.O. (2008). Reaching a Consensus in a Dynamically Changing Environment: A Graphical Approach. *SIAM Journal on Control and Optimization*, 47(2), 575-600.

2. Seneta, E. (1981). *Non-negative Matrices and Markov Chains.* (second ed.), New York: Springer-Verlag.

3. Horn, R.A., & Johnson, C.R. (1985). *Matrix Analysis.* New York: Cambridge University Press.
